# Effect of a *Saccharomyces cerevisiae* Postbiotic Feed Additive on *Salmonella* Enteritidis Colonization of Cecal and Ovarian Tissues in Directly Challenged and Horizontally Exposed Layer Pullets

**DOI:** 10.3390/ani13071186

**Published:** 2023-03-28

**Authors:** W. Evan Chaney, Hannah McBride, George Girgis

**Affiliations:** 1 Cargill, Inc., 15407 McGinty Road West, Wayzata, MN 55391, USA; 2 Nevysta Laboratory, Iowa State University Research Park, Ames, IA 50010, USA

**Keywords:** layers, *Salmonella*, postbiotic, pre-harvest food safety, additives, antibiotic alternative, intervention

## Abstract

**Simple Summary:**

In an era of increasingly complex global trade, continual evolution of production practices, and emerging threats from antibiotic resistance, new strategies for maintaining the health and performance of poultry flocks remain critical along with reducing risk from foodborne pathogens such as *Salmonella* Enteritidis (SE). Natural feed-additive technologies, often referred to as antibiotic alternatives, may play a key role. In this study, layer pullets were fed a control diet with or without a postbiotic feed additive and subsequently challenged directly or indirectly with SE at 16 weeks of age to evaluate the effect of the postbiotic for preventing or reducing SE colonization loads in birds directly or indirectly exposed to the pathogen. Within birds indirectly exposed to the SE inoculation, the postbiotic was associated with a significant reduction of SE-positive individual birds and their associated SE loads when compared to the control birds 7 days after inoculation, with non-significant yet explorable outcomes after 14 days post-challenge. These data support previous research findings in the literature indicating the postbiotic feed additive may aid in reducing SE in poultry production and may therefore be a candidate component of a comprehensive pre-harvest food-safety management plan.

**Abstract:**

Determining the efficacy of feed-additive technologies utilized as pre-harvest food-safety interventions against *Salmonella enterica* may be influenced by factors including, but not limited to, mechanism of action, experimental design variables, *Salmonella* serovar(s), exposure dose, route, or duration in both controlled research and real-world field observations. The purpose of this study was to evaluate the dietary inclusion of a *Saccharomyces cerevisiae* fermentation-derived postbiotic (SCFP) additive (Diamond V, Original XPC^®^) on the colonization of cecal and ovarian tissues of commercial pullets directly and indirectly exposed to *Salmonella* Enteritidis (SE). Four hundred and eighty commercial, day-of-age W-36 chicks were randomly allotted to 60 cages per treatment in two identical BSL-2 isolation rooms (Iowa State University) with four birds per cage and fed control (CON) or treatment (TRT) diets for the duration of study. At 16 weeks, two birds per cage were directly challenged via oral gavage with 1.1 × 10^9^ CFU of a nalidixic-acid-resistant SE strain. The remaining two birds in each cage were thus horizontally exposed to the SE challenge. At 3, 7, and 14 days post-challenge (DPC), 20 cages per group were harvested and sampled for SE prevalence and load. No significant differences were observed between groups for SE prevalence in the ceca or ovary tissues of directly challenged birds. For the indirectly exposed cohort, SE cecal prevalence at 7 DPC was significantly lower for TRT (50.0%) vs. CON (72.5%) (*p* = 0.037) and, likewise, demonstrated significantly lower mean SE cecal load (1.69 Log_10_) vs. CON (2.83 Log_10_) (*p* = 0.005). At 14 DPC, no significant differences were detected but ~10% fewer birds remained positive in the TRT group vs. CON (*p* > 0.05). These findings suggest that diets supplemented with SCFP postbiotic may be a useful tool for mitigating SE colonization in horizontally exposed pullets and may support pre-harvest food-safety strategies.

## 1. Introduction

Human salmonellosis is a disease well recognized to be partially attributable to foodborne vectors for which poultry and poultry products contribute to global incidence. For decades, the poultry industry has implemented and continually advanced preventative control measures and processing aids or interventions targeting the mitigation of foodborne pathogens such as *Salmonella enterica* [1,2,3,4]. Despite great advancements and effort, salmonellosis remains a recognized challenge for the poultry industry. As regulatory requirements and consumer desires continue to influence the evolution of industry production practices globally, food safety will remain a critical focal point.

In the United States alone, the Interagency Food Safety Analytics Collaboration (IFSAC) reported 811 *Salmonella* outbreaks between 1998 and 2017. Of these outbreaks, illnesses attributable to chicken products and eggs constituted 14.0% and 7.9% of attributable outbreaks, respectively. In the most recent reporting years, attribution to eggs has continued a downward trend to 6.9% (2018), 6.3% (2019), and 5.7% (2020) as consumption per capita has continued to increase, demonstrating continued industry progress [5,6]. *Salmonella enterica* serovar Enteritidis has long been primarily associated with table egg consumption, which led the U.S. Food and Drug Administration to implement the Egg Safety Rule in 2009 [7]. Vaccination, biosecurity, and farm-management practices, as well as egg-sanitization technologies, have largely been the primary controls for *Salmonella* risk management in the egg industry [8,9]. Despite these measures’ collective success, cases of human salmonellosis remain attributable to egg consumption necessitating the continuous improvement and implementation of novel food-safety solutions in the live-production environment. 

Specific intervention against foodborne pathogens in the live-production environment has historically been managed via biosecurity practices and vaccination, generally, with live, attenuated strains of *Salmonella* serovars such as Typhimurium, Enteritidis, or a combination of targeted serovars [10]. Animal feed and water provide excellent candidacy as carriers of intervention technologies targeting foodborne pathogens as they are consumed by the animal population consistently over the duration of rearing. Current feed-additive technologies, often referred to as natural alternatives to antibiotics, consist of products that target measurable improvements in health and performance of the animals and often do not require withdrawal periods, allowing for continual administration in the feed and water. Many such products are classified as “biotics” and include prebiotics, probiotics, synbiotics, or postbiotics, as described by the International Scientific Association of Probiotics and Prebiotics (ISAPP). Other examples may be of phytogenic origin [11,12,13,14]. Each of these categories of product types may benefit aspects of host health and performance through diverse mechanisms of action, some of which may offer additional efficacy as pre-harvest interventions against various pathogens [15,16,17,18,19]. 

Postbiotics are a more recent ISAPP defined category described as “a preparation of inanimate microorganisms and/or their components that confer a health benefit on the host” [12]. Similarly, postbiotics have also been described as “the bioactive compounds resulting from fermentation processes by food-grade microorganisms” [20]. Original XPC^®^ (SCFP; Diamond V, Cedar Rapids, IA, USA) is a postbiotic product consisting of bioactive compounds derived from a proprietary *Saccharomyces cerevisiae* fermentation process. Reported health and performance benefits of feeding SCFP in poultry have included lower corticosterone in response to environmental stressors, improved heterophil/lymphocyte ratios and physical asymmetry during stress events, reduced intestinal lesions and improved immune function during *Eimeria maxima* and *E. tenella* infection, and improved feed conversion, growth, meat yield, and egg production [21,22,23,24,25,26,27,28,29]. 

Use of SCFP as a pre-harvest intervention for reducing the colonization of *Salmonella enterica* in poultry has been recently reported in both broiler and layer chickens. In a longitudinal study, commercial broilers fed SCFP on a Honduran farm demonstrated significant reductions in *Salmonella enterica* cecal prevalence and loads when compared to a cohort of flocks fed a standard diet without SCFP inclusion [30]. The ability of SCFP to reduce the colonization potential of *Salmonella* Enteritidis in experimentally challenged layer chickens has also been recently described [31,32,33]. The efficacy of feed-additive technologies, such as SCFP postbiotic, to reduce the colonization potential of *Salmonella enterica* may be influenced by a variety of factors including, but not limited to, additive mechanism of action, bird genetics and age, *Salmonella* serovar(s), exposure dose or route, exposure duration, feed composition, analytical sample type, or collection timepoint in both controlled and real-world research. Therefore, the purpose of this study was to evaluate the effectiveness of feeding SCFP postbiotic to reduce *Salmonella* Enteritidis colonization potential in layer pullets challenged directly and indirectly at 16 weeks of age. 

## 2. Materials and Methods

**Animal Husbandry and Experimental Design.** Six hundred, day-old W-36 layer chicks (Hy-line North America, LLC, Warren, IN, USA) were procured without *Salmonella* vaccination from the supplier and randomly divided into two groups assigned to separate, identical BSL-2 isolation rooms at the Laboratory Animal Resources (LAR) isolation facility of Iowa State University (ISU) in Ames, IA, USA. All rearing and experimental procedures were reviewed and approved by the Institutional Biosafety Committee and Institutional Animal Care and Use Committee of the Iowa State University system (IACUC #22-035). Each experimental group of pullets was reared in single-tier cage units measuring 30″ (W) × 240″ (L) × 18″ (H) until the age of 7 weeks. Chick papers were used on cage floors in the first 9 days and then removed. Each cage unit was equipped with feeders and drinking nipples as recommended by the birds’ supplier and in accordance with the Guide for the Care and Use of Agricultural Animals in Research and Teaching [34]. Temperature and humidity were automatically controlled and adjusted according to the recommendations of the birds’ supplier. Artificial light was evenly distributed and turned on and off using an automatic timer. The automatic timer was programmed according to the recommendation of the birds’ supplier during the acclimatization and in-life experimental period.

At the age of 7 weeks, pullets were assigned to 3-tier cage units. Each cage measured 15ʺ (W) × 30ʺ (L) × 18ʺ (H) cage (n = 120) and 2 pullets were randomly assigned to be directly challenged and 2 served as horizontally exposed contacts. Extra cages were populated to account for unforeseen mortality (n = 30 cages/n = 120 pullets). Pullets in each cage shared a single feeder and a single water nipple. Pullets received *Salmonella* and had manure collection trays underneath. Four (4) pullets were placed in each Enteritidis (SE) challenge 9 weeks after moving to the 3-tier experimental cage units, during which experimental groups were fed appropriate diets with or without the SCFP postbiotic (Table 1).

**Treatment Diets.** Pullets were fed ad libitum with all-vegetarian mash rations formulated to meet the nutritional recommendations by the birds’ supplier. The same feed formulation, with or without the SCFP test item, was provided as assigned to each experimental group and age. To account for absence of SCFP in the CON diets, 2.5 lbs./ton additional ground corn were added. Feed milling was conducted at the Iowa State University Department of Animal Science feed mill. Routine bacteriological analyses were conducted on each batch of the feed used in the study to verify absence of SE. Feed samples from each batch manufactured were collected at time of feed mixing and shipped to the SCFP postbiotic manufacturer (Diamond V, Cedar Rapids, IA, USA) for tracer recovery testing to verify the proper inclusion rate of 2.5 lb./ton SCFP postbiotic in each diet phase. Pullets were provided with fresh potable water ad libitum (Table 2).

**SE-Challenge Preparation and Administration.** Preparation and quantification of the inoculum was completed according to Nevysta Laboratory standard procedures. Briefly, a loopful of colonies was transferred to tryptic soy broth (TSB) and incubated at 37 °C (shaker incubator) overnight. A 1:10 dilution was further incubated in a shaker incubator to prepare the challenge inoculum. Determination of the inoculum concentration was completed using optical density measurements at 600 nm. The inoculum was harvested at an optical density indicative of a concentration of 10^9^ CFU/mL. Bacterial cells were pelleted (15 min, 4 °C, 5000 rpm) and washed twice, resuspended in sterile deionized water, and used immediately. A sample of the inoculum was subject to serial dilution to determine the actual SE concentration in the inoculum using the standard plate-count method onto XLT-4 agar. The concentration of the challenge dose was verified, and the actual count was found to be 1.1 × 10^9^ CFU/dose. Challenge doses were administered orally once to pullets at the age of 16 weeks using a dosing syringe and gavage tube, with each directly challenged pullet received 1 mL of inoculum containing 1.1 × 10^9^ CFU of nalidixic-acid-resistant SE strain.

**Sample Collection.** Upon chick placement and at 14 weeks of age, environmental swabs were collected from chick papers or the droppings in cage unit trays and tested to ensure no detectable wild-type SE infection prior to challenge. Each swab was placed in a sterile sampling bag and gloves were changed between samples. At 6 days post-challenge (DPC), environmental swabs were collected as described above to verify SE shedding associated with the experimental infection. Eighty pullets (20 cages) from each group were humanely euthanized by cervical dislocation at 3, 7, and 14 DPC (Table 1). Cecal pouches and ovaries were aseptically collected from individual birds and transported to the laboratory on ice packs for immediate sample preparation and microbiological analysis.

***Salmonella* Analysis.** Environmental swabs were processed for *Salmonella* isolation using pre-enrichment in buffered peptone water, secondary enrichment in tetrathionate Hajna (TTH) broth and plating on xylose lysine tergitol-4 (XLT-4) agar and Brilliant Green with Novobiocin (BGN) agar (Becton Dickinson, Sparks, MD). Suspected colonies were further tested in triple sugar iron (TSI) and lysine iron (LIA) slants (Becton Dickinson, Sparks, MD) followed by serogrouping using appropriate O and H *Salmonella* antisera (SSI Diagnostica, Hillerød, Denmark). 

The contents of cecal pouches were aseptically squeezed into sterile conical tubes and weighed. Sterile saline was added at a ratio of 1:10 weight per volume. Ten-fold serial dilutions were prepared, and the standard plate count method was conducted using XLT-4 agar plates containing 25 μg nalidixic acid/mL. Plates were incubated aerobically for 24 hr at 37 °C and morphologically typical *Salmonella* colonies were counted. Numbers of *Salmonella* were calculated by the following formula:

CFU/g = (Number of colonies × dilution factor)/volume cultured

Randomly selected colonies from positive countable plates were serologically confirmed to be the SE-challenge strain to validate the accuracy of visual counts. Samples with SE counts below the detection limit were subject to enrichment and culture isolation to determine the absence or presence of SE.

Ovaries were homogenized in peptone water using a stomacher and then incubated aerobically for 24 hr at 37 °C. Enrichment was performed by transferring incubated samples to TTH broth at a ratio of 1:10 volume to weight and incubation at 42 °C for 24 hr. Incubated media were streaked on XLT-4 agar plates containing 25 μg of nalidixic acid/mL. Suspected colonies were further tested in TSI and LIA slants followed by serogrouping using appropriate O and H *Salmonella* antisera.

**Statistical Analysis.** All data were analyzed in SAS Version 9.4 (SAS Institute, Cary, NC). For cecal samples qualitatively positive for SE but non-enumerable by plate count, the method limit of quantitation was assigned for statistical analyses (100 CFU/g) and all quantitative estimates were log_10_ transformed prior to analysis. Quantitative and qualitative SE outcomes were modeled using PROC GLIMMIX with fixed effects of treatment, day, and challenge status with the random effect of cage. LS means were computed and pairwise comparisons determined significantly different at *p* < 0.05.

## 3. Results

All environmental swabs collected from experimental groups prior to challenge administration at 16 weeks tested negative for the presence of SE. All environmental swabs collected 6 days post-challenge administration tested positive for SE, confirming shedding at the cage-level associated with established infection.

For directly challenged birds, there were no significant differences in cecal prevalence between CON and SCFP postbiotic at 3 DPC (97.5 vs. 100%), 7 DPC (97.5 vs. 97.5%), or 14 DPC (90 vs. 80%). For ovary tissues, there were also no significant differences detected at 3 DPC (35% vs. 40%), 7 DPC (30 vs. 32.5%), or 14 DPC (10 vs. 2.5%). At 14 DPC, the SCFP postbiotic directly challenged cohort were observed to have 10% less ceca-positive individuals as compared to CON (*p* = 0.21) and 7% fewer positive ovaries (*p* = 0.32), but these observations were not statistically significant (Table 3 and Table 4).

Conversely, for indirectly challenged birds (those horizontally exposed to SE by cage mates/environment), the SCFP postbiotic cohort ceca were significantly lower for SE prevalence (50% vs. 72.5%; *p* = 0.037) at 7 DPC when compared to CON, despite equivalence at 3 DPC (45% vs. 40%). Notably, as similarly observed within the direct challenge cohort, the SCFP-treated birds again were observed to have 10% fewer positive individuals at 14 DPC (42.5% vs. 52.5%), though not statistically significant (*p* = 0.36). No differences were observed between treatment cohorts for SE prevalence in ovary tissues of indirectly challenged birds (Table 3 and Table 4). 

**Figure 1:**
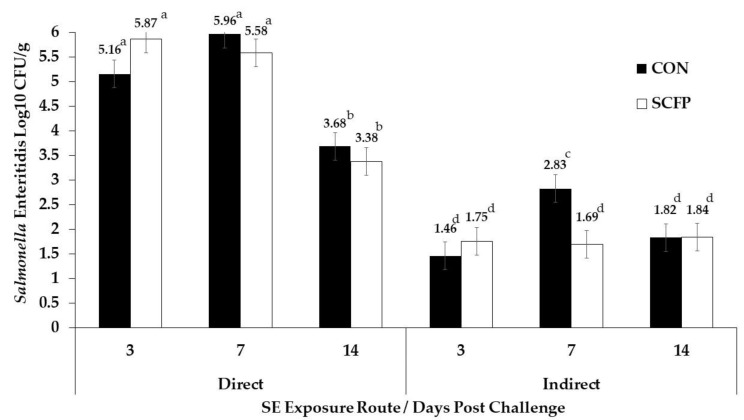
LS means with standard error for SE load in the ceca of directly challenged and indirectly exposed pullets. Different superscript letters denote significant differences between days and treatments (*p* < 0.05).

Mean log_10_ CFU/g SE load estimates in the ceca of directly challenged birds between CON and SCFP postbiotic cohorts at 3 DPC (5.16 vs. 5.87 Log_10_ CFU/g), 7 DPC (5.96 vs. 5.58 Log_10_ CFU/g), and 14 DPC (3.68 vs. 3.38 Log_10_ CFU/g), respectively, were statistically equivalent. The observed mean estimates were, however, observed to be ~0.3 to 0.4 Log_10_ CFU/g lower than CON at 7 and 14 DPC (Figure 1) and mean estimates for the SCFP-fed birds reduced across sampling points whereas the CON birds numerically increased at 7 DPC when compared to 3 DPC. 

Within the indirectly challenged birds, a significant 1.13 Log_10_ CFU/g reduction benefit for the SCFP postbiotic fed cohort was observed over the CON cohort for mean log_10_ CFU/g SE load in ceca at 7 DPC (1.70 vs. 2.83 Log_10_ CFU/g; *p* < 0.001). No significant differences were observed at 3 DPC (1.46 vs. 1.76 Log10 CFU/g) or 14 DPC (1.82 vs. 1.84 Log_10_ CFU/g) between CON and SCFP. Although similar to the directly challenged CON birds, mean Log_10_ CFU/g SE load in the indirect CON cohort significantly increased by 1.37 Log_10_ CFU/g (*p* = 0.0009) from 3 DPC to 7 DPC, whereas mean load in the indirect SCFP postbiotic cohort remained stable with fewer qualitatively positive individuals contributing overall to the mean estimate (Table 2). 

## 4. Discussion

*Salmonella* Enteritidis is a foodborne pathogen of significant public health concern and its association with the poultry meat and egg industries has been well documented. Therefore, feed-additive technologies that may additionally function as pre-harvest food-safety interventions, while improving the health and performance of the bird, could contribute to the stepwise, multi-hurdle reduction of pathogen risk. While often referred to as natural antibiotic alternatives, many feed-additive technologies do not specifically target *Salmonella* through a well characterized bacteriostatic or bactericidal mechanism. Rather, many additive technologies influence the hosts’ immune system, microbiome, or other host-specific attributes which may indirectly be associated with reducing the colonization potential of *Salmonella* or other pathogens. Prebiotics and probiotics are largely targeted to influence composition of the microbiome thereby favoring production of metabolites such as bacteriocins which may be antagonistic to *Salmonella* or, alternatively, by competitively excluding pathogens in the gut environment through niche resource utilization or other pathways [35,36]. The SCFP postbiotic is a metabolite-rich, complex product with multifunctional benefits recognized to modulate host immunity and the microbiome. Its ability to reduce the colonization potential of pathogens may be multi-factorial. As an example, SCFP increases volatile fatty acid production. Fatty acids not only contribute to host epithelial-cell health but are also antagonistic to *Salmonella* [27,37,38,39]. Because of host, environment, and pathogen level variables, modeling the efficacy of feed-additive technologies in vivo is necessary and can be influenced by experimental design and sampling strategies. Therefore, additive technologies should ideally demonstrate efficacy under a variety of controlled and real-world conditions. 

Recent literature demonstrates the variable efficacy of feed-additive technologies in the reduction of *Salmonella enterica*, and more specifically serovar Enteritidis, in layer-type chickens. In a study evaluating a commercial yeast cell wall (YCW) preparation fed from day 1 to 17 weeks of age and week 10 to 17 weeks of age in layer pullets, authors reported no reductions in cecal or ovary tissue SE prevalence (% SE-positive) for any treatment at 7 DPC. Adjusted mean log_10_ MPN/g SE load reductions ranged from 1.2 to 1.4 log_10_ MPN/g in the ceca of YCW groups, and approached significance, but were not statistically significant. In this research, a large proportion of samples (across treatments) exceeded the upper quantitative limit of the most probable number enumeration method, for which authors noted the high dose direct SE challenge (1.8 × 10^9^ CFU) may have been too overwhelming to assess cecal colonization protection by the additive [40]. In another study evaluating commercial YCW delivered via water from 1 to 42 days of age, broilers directly challenged with SE at 28 days (3.0 × 10^8^ CFU) had significant mean log_10_ CFU/g SE reductions of 0.85 and 0.76 in feces collected at 7 and 14 DPC, respectively, when compared to control birds [41]. A commercial YCW product evaluated alone and in combination with a commercial *Bacillus* spp. probiotic was compared to SCFP postbiotic and controls in 9-week-old pullets directly challenged with SE (3.0 × 10^7^ CFU) after 3 days of treatment. Results indicated no reduction in SE prevalence for any treatment but significant mean log_10_ MPN/g SE reductions of 0.79 and 0.86 for the probiotic and SCFP postbiotic treatment groups, respectively, at 7 DPC. The YCW product alone, however, was equivalent to the control birds [33]. In a similar model evaluating YCW alone and in combination with a *Bacillus* probiotic, 56-week-old laying hens were orally challenged at 60 weeks with SE (7.0 × 10^7^ CFU) and sampled 7 DPC. Again, the authors reported no SE prevalence reduction in ceca, but in this case, reported significant mean log_10_ MPN/g reductions for YCW and probiotic treatments alone, approximating adjusted means of 1.4 log_10_ MPN/g, but only 0.78 log_10_ MPN/g for the combined product group which was not significantly different from control [42]. The effects of a treatment containing a commercial YCW combined with a *Bacillus subtilis* probiotic administered to day-old layer chicks and directly challenged at 8 days with SE (2.1 × 10^9^ CFU), resulted in mean cecal log_10_ CFU/g reductions of 0.61, 0.49, 0.45, and 1.25 at 3, 6, 10, and 14 DPC, respectively [43].

The model reported herein utilized a mixed SE challenge, allowing for treatment efficacy comparison in both directly and horizontally exposed cohorts of birds. Similar to research described above, a high-dose direct challenge of SE (1.1 × 10^9^ CFU) was administered to the direct-challenge cohort in our study and there were no statistically significant differences between CON and SCFP postbiotic for SE prevalence or load in the cecal or ovary tissues, although observed mean log_10_ CFU/g were slightly less than CON for the SCFP postbiotic-fed cohort. As directly challenged birds shed SE, horizontally exposed birds were, therefore, subject to increasing exposure doses, and notably, SE cecal prevalence in the horizontally exposed SCFP postbiotic fed cohort demonstrated a significant 22.5% reduction (31% improvement over control) and greater than 1.0 log_10_ CFU/g load reduction at 7 DPC compared to the CON, suggesting that SCFP imparted a protective effect against SE colonization potential as exposure doses and pressure increased from the directly challenged birds. Enumeration of SE following an experimental challenge is an indication of the potential effect of any given treatment on *Salmonella* shedding at the time of sampling only. The frequency of shedding is known to decline steadily after artificial challenge, however, persistent colonization is evident in either directly or horizontally exposed chickens in some studies [44]. By 14 DPC, SE cecal load estimates were equivalent, however, it is notable that despite similar mean load estimates, 10% fewer positive individuals were contributing in the SCFP postbiotic cohort. This observation of fewer SE-positive individuals, in both the directly and horizontally exposed cohorts, might suggest that some birds were able to effectively clear the challenge faster than those not fed SCFP postbiotic. Gast and colleagues noted that the probability of persistent infection may involve a subtle interplay between host susceptibility, the challenge strain, and the dose of SE [45]. The combined observations and data from the current study suggest that SCFP postbiotic imparts a benefit to the host wherein SCFP-fed birds were less susceptible to high colonization loads and overall persistence of artificial SE infection at variable doses. This warrants further research which could evaluate the colonization potential and shedding duration of individuals fed SCFP postbiotic and challenged with variable doses or strains of SE. In extrapolating to commercial applications, reducing the number of positive individuals and their associated shedding loads may reduce overall horizontal transmission potential within a confined production population.

In a very similar study design evaluating a commercial YCW product in 16-week-old pullets challenged with SE (1.7 × 10^9^ CFU), no differences were observed in cecal SE prevalence between treatments at 7 DPC in directly or indirectly challenged cohorts, however, a significant reduction in mean SE load by 1.0 log_10_ CFU/g was observed in the directly challenged birds fed YCW [46]. Interestingly, the mean SE cecal loads in the directly challenged and indirectly exposed CON fed birds at 7 DPC was >2.2 log_10_ CFU/g lower in the previously described study when compared to our study despite a very similar challenge dose, thus suggesting that the challenge uptake or retention was more severe or elevated in our study. A limitation of these and many in vivo pathogen challenge models is the ability to truly verify day 0 treatment equivalence in the pathogen challenge. In the current study at 3 DPC the SCFP birds were observed to have slightly elevated mean log_10_ CFU/g SE cecal loads though not significantly so. This observation could indicate that 3 DPC is simply too soon to evaluate an effect after a high-dose artificial challenge or could be an artifact of another factor as it is more common in the literature to observe sampling timepoints of 5 or 7 DPC for similar studies. Alternatively, the lower observed prevalence and mean SE load at 7 and 14 DPC in the SCFP postbiotic cohort could suggest an overall faster rate of SE clearance to non-detectable levels as previously discussed. Future work could consider fecal sampling or cloacal sampling over consecutive days prior to organ harvest to demonstrate or adjust for relative shedding loads. Regardless, the two studies highlight that challenge dose appears to be a key consideration, particularly when comparing trial outcomes. Comparative evidence from studies utilizing lower SE direct challenge doses suggests that dose is a key variable between studies. Gingerich and colleagues evaluated SCFP postbiotic in layer pullets fed from 1–32 days and directly challenged with SE (1.0 × 10^6^ CFU) on day 28, reporting no significant differences in cecal prevalence but a significant 1.14 log_10_ CFU/g mean SE cecal load reduction at 7 DPC (*p* < 0.0001) [32]. At 9 weeks of age, birds fed SCFP postbiotic and directly challenged with SE (3.0 × 10^7^ CFU) demonstrated a 0.86 log_10_ MPN/g reduction over controls at 7 DPC [33]. When compared to a *Pediococcus acidilactici* probiotic, antibiotic and control treatment groups in hens reared from 44–57 weeks and directly challenged at 53 weeks with SE (1.0 × 10^7^ CFU), the SCFP -fed cohort demonstrated a significant 1.0 log_10_ CFU/g mean SE reduction (*p* < 0.05) over the control and was significantly lower than the probiotic- and antibiotic-fed treatment groups at 5 weeks post-challenge [31]. These studies demonstrating SE load reduction efficacy by SCFP postbiotic under direct-challenge conditions utilized SE-challenge doses that were approximately 2.0 + log_10_ CFU lower than the dose administered in the current study. This may have been a more appropriate dosage to evaluate effect in the directly challenged cohort and may have demonstrated even larger treatment separation in the horizontally exposed cohorts. 

These observations in the current and referenced studies suggest a combination of challenge dose, exposure model, study design, and host variables likely contribute to SE-challenge-dose retention and may influence the magnitude of treatment differentiation that is statistically distinguishable in such direct-challenge SE models evaluating natural feed-additive technologies. Dose and strain of SE have been associated with the persistence of fecal shedding in experimental models demonstrating a dose-response effect in detection of SE in feces or cloaca over time [45,47,48]. The difference in selection of a challenge model may be asking the question of any given additive’s ability to reduce a high-dose artificial infection (direct challenge) versus the ability to modify host factors to prevent colonization and propagation when exposed, horizontally, to transient and variable SE doses (indirectly exposed). The latter may arguably be more akin to real-world transmission dynamics and therefore more applicable to industry practice. 

## 5. Conclusions

In an era of increasingly complex global trade and continual evolution of production practices, maintaining the health and performance of poultry flocks is critical and natural feed-additive technologies may play a vital role. The present study demonstrates the ability of SCFP postbiotic to influence and reduce *Salmonella* Enteritidis in the ceca of horizontally exposed birds at 7 days post-challenge with *Salmonella* Enteritidis with non-significant yet explorable outcomes after 14 days post-challenge.. Inclusion of feed additives, such as SCFP postbiotic, in the production diets of poultry flocks may be an effective intervention step in a comprehensive pre-harvest food-safety management plan.

## Figures and Tables

**Table 1:** Experimental design overview.

Group	SCFP Inclusion	SE Challenge	Number of Birds	Number of Cages	Total Number of Birds/Treatment
Treatment	+	Direct	120	60	240
		Indirect	120		
Control	−	Direct	120	60	240
		Indirect	120		

SE-negative pullets were reared under BSL-2 isolation and fed appropriate experimental diets starting from day 0. Four pullets were assigned to each of 60 cages per group and at 16 weeks, two randomly selected per cage for direct-challenge via oral gavage with a nalidixic-acid-resistant *Salmonella* Enteritidis isolate dose of 1.1 × 10^9^ CFU/bird. The two remaining birds were indirectly exposed to the SE challenge via a shared environment. At 3, 7, and 14 days post-challenge, 20 cages (*n* = 80 pullets) were sampled from each experimental group for SE enumeration and isolation from cecal pouches and ovaries.

**Table 2:** Basal diet formulations (lbs./ton) for starter and grower rations without (CON) and with test-article inclusion at milling (SCFP).

Ingredient	0–7 Weeks Starter (20.3% Protein)	8–18 Weeks Grower (18.0% Protein)
Ground corn	1137.52	1269.63
Soybean meal	670.87	561.91
Calcium carbonate	38.93	34.71
21% monosodium phosphate	35.76	34.43
Salt	10.05	9.35
Soybean oil	78.19	69.51
Choline chloride	1.13	1.21
DL methionine	9.47	3.12
Lysine	13.59	11.64
Vitamin mix	2.00	2.00
Test article (SCFP)—test diet only	2.50 *	2.50 *
Total (lbs.)	2000	2000

* Not included in CON diet. The CON diet was allotted 2.5 lbs. /ton additional ground corn in the formulation to adjust for absence of Test Article.

**Table 3:** Prevalence and number of SE-positive ceca in pullets directly challenged and indirectly exposed to the challenge strain of SE.

Days	Direct Challenge		Indirect Challenge	
Post-Challenge	CON	SCFP	CON	SCFP
3 DPC	97.5% (39/40) ^a^	100% (40/40) ^a^	40.0% (16/40) ^b^	45.0% (18/40) ^b^
7 DPC	97.5% (39/40) ^a^	97.5% (39/40) ^a^	72.5% (29/40) ^b^	50.0% (20/40) ^c^
14 DPC	90.0% (36/40) ^a^	80.0% (32/40) ^a^	52.5% (21/40) ^b^	42.5% (17/40) ^b^

Prevalence of SE in the ceca of directly challenged and indirectly exposed pullets following oral gavage of 1.1 × 10^9^ CFU at 16 weeks of age. Values represent the number of SE culture positive birds/total number of birds sampled. Values with different superscripts within a row are significantly different (*p* ≤ 0.05).

**Table 4:** Prevalence and number of SE-positive ovaries in pullets directly challenged and indirectly exposed to the challenge strain of SE.

Days	Direct Challenge		Indirect Challenge	
Post-Challenge	CON	SCFP	CON	SCFP
3 DPC	35.0% (14/40) ^a^	40.0% (16/40) ^a^	2.5% (1/40) ^b^	2.5% (1/40) ^b^
7 DPC	30.0% (12/40) ^a^	32.5% (13/40) ^a^	5.0% (2/40) ^b^	5.0% (2/40) ^b^
14 DPC	10.0% (4/40)^a^	2.5% (1/40) ^a^	0.0% (0/40) ^a^	2.5% (1/40) ^a^

Prevalence of SE in the ovaries of directly challenged and indirectly exposed pullets following oral gavage of 1.1 × 10^9^ CFU at 16 weeks of age. Values represent the number of SE-culture-positive birds/total number of birds sampled. Values with different superscripts within a row are significantly different (*p* ≤ 0.05).

## Data Availability

Observed microbiological data obtained from analytical measurements as described are reported within the article. Restrictions may apply to the availability of some additional information due to propriety, but requests may be forwarded to the corresponding author.
